# Panelphobia and Its Cure

**Published:** 1922-12

**Authors:** R. W. Harris

**Affiliations:** (formerly Assistant Secretary, responsible for the administration of National Health Insurance Medical Benefit, Ministry of Health)


### PANELPHOBIA AND ITS CURE.

By R. W. HARRIS (formerly Assistant Secretary, responsible for the administration of National Health Insurance Medical Benefit, Ministry of Health).

ONE of the symptoms of hydrophobia is a horror of water; the disease derives its name from it. Similarly, panelphobia may be said to spring from a horror of the panel system, sometimes felt but more often simulated. It flourishes on the platform and in other places where men and women assemble in large numbers, and it can often be effectively treated simply by turning off the limelight. It fails, generally, to make much headway in the home.

I am moved to reflect on this matter by the recent shower of newspaper cuttings which indicate that the season of conferences has been with us and that symptoms of panelphobia have manifested themselves in the usual fashion. It is the duty of everyone who is interested in the question of improving the health conditions of his native land to try and check these manifestations, and I make no apology for insisting that the National Health Insurance scheme of medical benefit is not going to be improved by outbreaks of panelphobia. The trouble with this complaint is that the doctors can do nothing to cure it. The great majority of doctors may continue to give to their patients the best of which they are capable. If they are not giving their best, they may, under certain conditions—shall we say, the stimulus of a fair and reasonable criticism in the place of the nagging kind, or freedom from constant upheavals in the conditions of their service and their pay ?—give an improved service which does represent their best. There will still remain to be dealt with the minority of unsatisfactory doctors who neglect their patients.

In an article in the "Yorkshire Evening Post" on October 27, Dr. Harry Roberts, who practises in the East End of London, very properly points out that "the panel system has not only no responsibility for the creation of this neglect, but actually provides the only efficient remedies for it. . . . It has the great merit of placing the remedy largely in the hands of the patient himself." The doctor's income, as Dr. Roberts goes on to point out, depends on the number of patients who select him, and, having selected him, remain with him. "It is up to the insured persons, by a mere process of selection, to eliminate from the ranks of panel doctors the dwindling minority of rotters and slackers—*the only ones that gain publicity.*"

Dr. Cox, the Medical Secretary of the British Medical Association, appeals, in the columns of the "Western Mail, on October 25, as he has so often done elsewhere, to some of those who are so anxious to decry the insurance medical service, to "help those of us whose anxiety it is to improve it," and in that way do "a good stroke of business for the public." Dr. Cox points out that the insured person should exercise the power which he possesses of changing his doctor when not satisfied, and that, under a system of employing whole-time salaried doctors, some public body would choose his doctor for him. It rests, therefore, very largely with the patient himself to remedy the abuses which exist, by the simple process of going to another doctor. The friends of the insured person can do him a service by so advising him, and by further advising him to recommend others to change their doctor likewise; this method is much more effective than using a megaphone.

In the "Manchester Guardian" for October 19 Dr. Brennan, the honorary secretary of the Stockport Panel Committee, draws attention to the other remedy, that of bringing definite charges, supported by evidence, before the court of inquiry set up under the Regulations,—the Medical Service Sub-Committee of the Insurance Committee. "If I were reported in the Press," says Dr. Brennan, "as saying that lawyers robbed their clients or grocers adulterated their food, I should only arouse some resentment and some contempt for myself. But if I brought into a court of justice a fraudulent grocer or lawyer and had him punished on evidence of his guilt, then I should be doing a service to the community."

I do not think that there is any cause for satisfaction in learning that in a large County Insurance Committee area, as recently reported, there had been only one formal case of complaint in twelve months. It is obviously not due to the fact that every doctor in that county is giving a service free from criticism, but rather to the apathy of the insured persons and their friends. It is a weakness of the panel system that every doctor can undertake the service of treating insured persons until he is proved to have discredited the service. But it is a weakness easier to criticise than to remedy. To select the medical men who are to be entrusted with the treatment of the whole working population would involve conferring upon them some hall-mark of approval or recognition. Who will undertake it, and what shall be the test ? In the meantime, it is due to the service and all connected with it that, when cases of negligence occur, those who know the facts shall carefully prepare the case against the offending doctor and vigorously prosecute it to its conclusion. It is not to be supposed that you can improve the panel system merely by sending round a man with a bell.

Panelphobia can be cured, not by the doctor, but by the patient. The treatment is very simple : rest and quiet. The disease has the peculiarity that one person has all the symptoms, and feels no ill effects, while other inoffensive mortals suffer the consequences. It is the insured person who ultimately suffers. The good doctor must lose heart in his work to some extent, and this will react on the interest which he takes in his patients, if he is constantly being told that he is taking part in a rotten system. That is not a fair description of the panel system. Of course, it has its defects, but some constructive suggestions are needed in the place of the "hot air" which so often does duty for criticism.

I am quite sure that the remedy is the more effective use of the existing machinery. In the course of a little quiet research into the various meanings of the word "panel," I have come across one which indicates that it signifies a pad or packing beneath a saddle to protect the horse's back. To those who would substitute for the present system some closer system of control over the doctors, I would commend the foregoing definition; if the pad is removed, the horse may be stimulated at first when the saddle begins to rub, but it is going to prove expensive in the long run, and not a little uncomfortable for the rider.

